# Relationship of depression with empathy, emotional intelligence, and symptoms of a weakened immune system

**DOI:** 10.3389/fpsyg.2023.1250636

**Published:** 2023-10-27

**Authors:** Gloria Grases, Maria Antonia Colom, Pilar Sanchis, Felix Grases

**Affiliations:** ^1^Centro de Enseñanza Superior Alberta Gimenez (CESAG), Palma de Mallorca, Spain; ^2^Psychology Center (Juaneda Group Hospitals), Palma de Mallorca, Spain; ^3^University Institute of Health Sciences Research (IUNICS- IdisBa), University of Balearic Islands, Palma de Mallorca, Spain

**Keywords:** depression, empathy, emotional intelligence, immune system, patients

## Abstract

**Introduction:**

Previous studies have used different individual scales to examine the relationship of depression with emotional intelligence, empathy, and immune-based diseases. In this study, we used a combination of psychometric scales to examine the relationships of depression with emotional intelligence (intrapersonal and interpersonal), empathy (affective and cognitive), and symptoms of weakened immune system.

**Methods:**

This cross-sectional prospective study examined 158 volunteers (39 males and 119 females). A score of 10 or more on the Beck Depression Inventory-II (BDI-II) was used to define depression. The Cognitive and Affective Empathy Test (TECA) was used to assess empathy, and the Profile of Emotional Competence (PEC) was used to assess the self-perception of intrapersonal and interpersonal competence. The symptoms of a weakened immune system (WIS) were assessed by measurements of permanent tiredness, frequent infections and colds, slow wound healing, persistent and recurrent diarrhea, recurring herpes, insomnia and difficulty sleeping, and dry eyes.

**Results:**

The total PEC score and intrapersonal PEC score had negative correlations with depression, and the WIS score had a positive correlation with depression. The TECA score had no significant correlation with depression or the WIS score, but had positive correlations with the total PEC score, intrapersonal PEC score, and interpersonal PEC score.

**Conclusion:**

The total PEC score, intrapersonal PEC score, and WIS score were significantly associated with depression. The TECA score was not significantly associated with depression or the WIS score. Our findings suggest that improving intrapersonal emotional skills may improve function of the immune system and reduce the symptoms of depression. We suggest that further studies examine the effect of targeted improvement of interpersonal skills (empathy) on depression.

## 1. Introduction

Previous studies have used different approaches to examine the relationships of depression with immune-based diseases (Salim, 2016; Drevets et al., 2022). There is increasing evidence that a depressed mood and a state of sustained depression negatively influence the immune system (Sirera et al., 2006). Other studies showed that a high level of anxiety and depression were associated with clear increases in oxidative stress (Halliwell, 2006; Bouayed et al., 2009; Hovatta et al., 2010; Maes et al., 2011; Scapagnini et al., 2012; Grases et al., 2014), and that increased oxidative stress may contribute to the pathogenesis of multiple diseases (Kovacic and Somanathan, 2012). On the other hand, there is also evidence that pro-inflammatory cytokines can induce depression in patients who previously had no symptoms of mental disorders (Dantzer et al., 2008; Ramírez et al., 2018). Therefore, it seems that depression may lead to the development of certain diseases, and certain pathological states may lead to depression (Miller and Raison, 2016; Branchi et al., 2021).

A meta-analysis to study correlations between empathy and depression has been developed (Yan et al., 2021). Various studies have been analyzed, and results showed that empathy was not correlated to depression, however, a subgroup analyses found that while affective empathy and depression was significantly positively correlated, cognitive empathy was not. Moreover, interestingly the relationship between empathy and depression changed during development, with a positive correlation in adolescence and negative correlation in older adults (Yan et al., 2021). Some researchers consider empathy and depression as related (O’Connor et al., 2002; Berking and Wupperman, 2012; Schreiter et al., 2013; Tully et al., 2016), and some findings indicate that empathy is not always positively related to depression. In fact, the risk of depression is greater when there is excessive or insufficient empathy, or when empathy occurs in combination with other characteristics, such as low emotional self-regulation. In addition, even studies that have differentiated cognitive and affective empathy have not always had clear results on the relationship of empathy and depression. Some researchers proposed that cognitive and affective empathy may increase the risk of depression (Tone and Tully, 2014), although other studies suggested that cognitive and affective empathy had different roles in depression. There is also evidence that only affective empathy was positively related to depression (Gambin and Sharp, 2018; Calandri et al., 2019), and that cognitive empathy was negatively related to depression (Bennik et al., 2019). Another recent study carried out with children, demonstrated that high and low empathy were associated with increased depression, and moderate empathy was associated with the lowest level of depression (Cui et al., 2023).

Studies of the relationship between emotional intelligence and depression found a correlation between these two conditions (Żuchowicz et al., 2018), in that a high level of emotional intelligence was negatively related to depression (Doyle et al., 2021; Wells et al., 2021). An individual’s ability to perceive, evaluate, express, and regulate emotions is an important determinant of quality-of-life. A high level of emotional intelligence also appears to provide protection against mental disorders such as depression. Some research suggested that an individual experiences altered emotional regulation during the onset and maintenance of depression (Aldao and Nolen-Hoeksema, 2010). There are also relationships between depression and low emotional skills, such as inadequate emotional management (Nolidin et al., 2013), and between empathy and emotional intelligence (Bertram et al., 2016; Abe et al., 2018). A current study demonstrates that impaired emotional intelligence was correlated with symptoms of major depression and suicide attempts (Okasha et al., 2023). The relationship between emotional intelligence, sleep quality and depression were evaluated in a study which concluded that improving skills related to emotional intelligence can increase sleep quality and thus reduce depressive symptoms (Salguero-Alcañiz et al., 2021).

This study analyzed the possible associations among depression, emotional intelligence (intrapersonal and interpersonal), empathy (affective and cognitive), and symptoms of a weakened immune system in adults.

## 2. Materials and methods

### 2.1. Design

This is a cross-sectional prospective study.

### 2.2. Patient selection

One hundred and fifty-eight volunteers (39 males and 119 females), ranging in the age from 18 to 78 years, were consecutively recruited from the Psychology Center at Juaneda Group Hospitals (Balearic Islands, Spain) from January 2021 to June 2022. The inclusion criteria were diagnosis of a depressive disorder, marital conflict, or behavioral problem. The exclusion criterion was presence of any severe mental health disorder (e.g., schizophrenia or bipolar disorder). All participants were Caucasian and had medium-high social status. The minimum number of patients for detecting a 10% difference in WIS and PEC scores between patients with and without depression (considering 80% power and 95% confidence interval) was 60 patients per group (n = 120).

### 2.3. Psychometric instruments

Validated psychometric scales were used to evaluate the study participants. The Beck Depression Inventory-II (BDI-II; Beck et al., 2011) was used to assess the presence and severity of depression. This instrument consists of 21 multiple choice items, with each response scored on a Likert-type scale and has high reliability and adequate validity (example of an item from BDI-II “Guilty Feelings: 0. I do not feel particularly guilty; 1. I feel guilty over many things I have done or should have done; 2. I feel quite guilty most of the time; 3. I feel guilty all of the time”). A BDI-II score below 13 is considered normal (minimal depression), a score of 14 to 19 indicates mild depression, a score of 20 to 28 indicates moderate depression, and a score of 29 to 63 indicates severe depression. In this study, a BDI-II score of 10 or more was used to define depression.

The Cognitive and Affective Empathy Test (TECA) was used to assess skills related to knowing the thoughts, intentions, and emotions of others, and in sharing their emotional states (López-Pérez et al., 2008). This scale assesses empathic capacity from cognitive and affective approaches based on 33 items, with each response scored on a 5-point Likert scale and has four specific subscales (example of an item from TECA: “When a friend is sad, I get sad too”). The subscales of perspective taking (understanding the points of view of others) and emotional understanding (ability to recognize and understand the emotional states and intentions of others) assess cognitive empathy; the subscales of empathic stress (ability to share the negative emotions of another person, emotionally in tune with them) and empathic joy (ability to share the positive emotions and successes of others) assess affective empathy. This questionnaire has high validity and reliability (Supplementary Table 1).

The Spanish adaptation (Páez et al., 2016) of the Profile of Emotional Competence (PEC; Brasseur et al., 2013) was used to assess the self-perception of intrapersonal and interpersonal emotional competencies. This instrument has 50 items, each response is scored from 1 to 7 on a Likert-type scale (example of an item from PEC: “I can easily find the words to describe what I feel”). It assesses the ability to understand and identify emotions (their causes and consequences) and to regulate emotions (expressing them in a socially appropriate way and using them to guide thinking and behavior). This instrument has good reliability, structural validity, and criterion validity (Supplementary Table 2).

To assess the symptoms of weakened immune system (WIS), the presence (1 point) or absence (0 points) of seven specific symptoms were recorded: permanent tiredness, frequent infections and colds, slow wound healing, persistent and recurrent diarrhea, recurring herpes, insomnia and difficulty sleeping, and dry eyes. The total WIS score ranged from 0 to 7 points.

### 2.4. Statistical analysis

Data are presented as means and standard deviations, medians and interquartile ranges, or numbers and percentages, as appropriate. Cronbach’s alpha was calculated for each questionnaire as a measure of the internal consistency and reliability. Patients were divided in two groups according BDI-II score, three groups according to PEC score, and five groups according TECA score. Intergroup comparisons employed a one-way ANOVA, the Kruskal-Wallis test, the independent samples t-test, or the Mann Whitney U test for continuous variables, and the Chi-square test for categorical variables. Linear correlations (crude and adjusted) between BDI-II, WIS, total TECA, total PEC, intrapersonal PEC, and interpersonal PEC were calculated. Furthermore, an stepwise discriminant analysis were performed to construct a associative model for discrimination between individuals with and without depression.

Receiver operating characteristic (ROC) curves were used to determine the relationship of different factors with depression (BDI-II ≥ 10). The optimal cutoff values were determined by the Youden index (J), defined as sensitivity + specificity −1. Crude and adjusted odds ratios (ORs) of factors associated with depression were calculated relative to the reference of no depression (BDI < 10). A two-tailed *p*-value less than 0.05 was considered statistically significant. Statistical analyses were performed using SPSS version 23.0 (SPSS Inc., Chicago, IL, USA).

## 3. Results

### 3.1. Comparison of patients with and without depression

We examined 158 patients (Table 1). There were 119 females and 39 males, the average age was 40 ± 14 years, and there were 70 patients (44.3%) with depression (BDI-II ≥ 10) and 88 patients (55.7%) without depression (BDI-II, Cronbach’s alpha = 0.823).

**Table 1:** Characteristics of patients with and without depression.

	No depression (*n* = 88)	Depression (*n* = 70)	*p*-value
Age (years)	40 ± 15	40 ± 14	0.776
Gender (female)	63 (71.6%)	56 (80.0%)	0.267
BDI-II	8.7 ± 2.9	17.8 ± 3.8	<0.001
Severe	0 (0.0%)	2 (2.9%)	<0.001
Mild	0 (0.0%)	52 (74.3%)	
Moderate	0 (0.0%)	16 (22.9%)	
No depression	88 (100.0%)	0 (0.0%)	
WIS	2.1 ± 1.4	3.3 ± 1.2	<0.001
Permanent tiredness	34 (38.6%)	45 (64.3%)	0.001
Infections and colds	30 (34.1%)	31 (44.3%)	0.189
Slow healing	11 (12.5%)	19 (27.1%)	0.024
Diarrhea	12 (13.6%)	26 (37.1%)	0.001
Herpes	33 (37.5%)	28 (40.0%)	0.743
Insomnia	39 (44.3%)	46 (65.7%)	0.006
Dry eyes	27 (30.7%)	35 (50.0%)	0.014
PEC, total	186 ± 31	173 ± 28	0.005
Low	23 (26.1%)	29 (41.4%)	0.008
Medium	21 (23.9%)	23 (32.9%)	
High	37 (42.0%)	14 (20.0%)	
PEC, intrapersonal	96 ± 20	84 ± 19	0.001
Low	23 (26.1%)	33 (47.1%)	0.014
Medium	24 (27.3%)	18 (25.7%)	
High	34 (38.6%)	15 (21.4%)	
PEC, interpersonal	90 ± 16	91 ± 18	0.730
Low	25 (28.4%)	22 (31.4%)	0.817
Medium	25 (28.4%)	17 (24.3%)	
High	31 (35.2%)	26 (37.1%)	
TECA, total	62 ± 31	60 ± 32	0.617
Very low	4 (4.5%)	6 (8.6%)	0.721
Low	15 (17.0%)	9 (12.9%)	
Medium	18 (20.5%)	18 (25.7%)	
High	31 (35.2%)	22 (31.4%)	
Very high	17 (19.3%)	14 (20.0%)	
TECA, Adoption perspectives (AP)	56 ± 33	51 ± 32	0.425
TECA, Emotional understanding (CE)	67 ± 29	61 ± 33	0.291
TECA, Empathic stress (EE)	46 ± 30	54 ± 32	0.092
TECA, Empathic joy (AE)	65 ± 31	63 ± 31	0.567

Values are expressed as mean ± SD or *n* (%). *p*-value was calculated with student’s *t*-test for independent simples or Mann–Whitney U test (for quantitative data) or with chi-square or Fisher’s exact test (for qualitative data).

Analysis of the TECA scores in the total population indicated that 31 patients (19.6%) had very high scores, 52 (32.9%) had high scores, 36 (22%) had moderate scores, 24 (15.2%) had low scores, and 10 (63%) had very low scores (TECA, Cronbach’s alpha = 0.811). The total PEC results demonstrated that 51 patients had high scores (32.3%), 44 (27.8%) had medium scores, and 52 (32.9%) had low scores (PEC, Cronbach’s alpha = 0.815). The WIS results showed 79 patients (50.0%) had permanent tiredness, 61 (38.6%) had infections and colds, 30 (19.0%) had slow healing, 38 (24.1%) had diarrhea, 61 (38.6%) had herpes, 85 (53.8%) had insomnia, and 62 (39.2%) had dry eyes (WIS, Cronbach’s alpha = 0.644).

Relative to the group without depression, the group with depression had a higher total WIS score (3.3 ± 1.2 vs. 2.1 ± 1.4; *p* < 0.001), a lower total PEC score (173 ± 28 vs. 186 ± 31; *p* = 0.005), and a lower intrapersonal PEC score (84 ± 19 vs. 96 ± 20; *p* < 0.001). However, these two groups had no significant differences in total interpersonal PEC score or TECA score.

### 3.2. Comparison of patients with different PEC scores and TECA scores

We then analysed the characteristics of patients with low, medium, and high total PEC scores (Table 2). The group with the highest PEC score was older, had a lower BDI-II score, a lower WIS score, and a higher TECA score than the other two PEC groups.

**Table 2:** Characteristics of patients with different total PEC scores.

	Low	Medium	High	*p*-value for trend
	(*n* = 52)	(*n* = 44)	(*n* = 51)	
Age (years)	34 ± 15	43 ± 13	42 ± 14	0.006
Gender (female)	38 (73.1%)	30 (68.2%)	43 (84.3%)	0.189
BDI-II	14.8 ± 5.9	13.4 ± 4.1	10.4 ± 5.5	<0.001
Severe	2 (3.8%)	0 (0.0%)	0 (0.0%)	0.009
Mild	18 (34.6%)	20 (45.5%)	12 (23.5%)	
Moderate	9 (17.3%)	3 (6.8%)	2 (3.9%)	
No depression	23 (44.2%)	21 (47.7%)	37 (72.5%)	
WIS	3.0 ± 1.4	2.9 ± 1.5	2.2 ± 1.3	0.006
WIS permanent tiredness	33 (63.5%)	24 (54.5%)	21 (41.2%)	0.024
WIS infections and colds	22 (42.3%)	18 (40.9%)	18 (35.3%)	0.469
WIS slow healing	13 (25.0%)	8 (18.2%)	6 (11.8%)	0.084
WIS diarrhea	17 (32.7%)	12 (27.3%)	9 (17.6%)	0.082
WIS herpes	21 (40.4%)	22 (50.0%)	17 (33.3%)	0.473
WIS insomnia	28 (53.8%)	28 (63.6%)	23 (45.1%)	0.379
WIS dry eyes	22 (42.3%)	17 (38.6%)	19 (37.3%)	0.601
PEC, total	148 ± 18	183 ± 6	211 ± 15	<0.001
PEC intrapersonal	71 ± 13	91 ± 9	111 ± 13	<0.001
Low	44 (84.6%)	11 (25.0%)	1 (2.0%)	<0.001
Medium	8 (15.4%)	25 (56.8%)	9 (17.6%)	
High	0 (0.0%)	8 (18.2%)	41 (80.4%)	
PEC, interpersonal	79 ± 21	91 ± 9	101 ± 9	<0.001
Low	37 (71.2%)	9 (20.5%)	1 (2.0%)	<0.001
Medium	8 (15.4%)	20 (45.5%)	14 (27.5%)	
High	6 (11.5%)	15 (34.1%)	36 (70.6%)	
TECA, total	41 ± 31	64 ± 26	81 ± 21	<0.001
Very low	9 (17.3%)	1 (2.3%)	0 (0.0%)	<0.001
Low	15 (28.8%)	5 (11.4%)	1 (2.0%)	
Medium	16 (30.8%)	12 (27.3%)	8 (15.7%)	
High	7 (21.8%)	22 (50.0%)	20 (39.2%)	
Very high	5 (9.6%)	4 (9.1%)	22 (43.1%)	
TECA, Adoption perspectives (AP)	38 ± 32	51 ± 28	73 ± 27	<0.001
TECA, Emotional understanding (CE)	44 ± 31	69 ± 28	84 ± 19	<0.001
TECA, Empathic stress (EE)	44 ± 31	51 ± 33	53 ± 30	0.150
TECA, Empathic joy (AE)	50 ± 33	67 ± 31	77 ± 23	<0.001

Values are expressed as mean ± SD or *n* (%). *p*-values for trend were calculated using the total PEC score as a continuous variable in the correlation analysis.

Analysis of the five groups of patients with different TECA scores showed that the group with the highest cognitive and an affective empathy had higher total intrapersonal and interpersonal PEC scores than the other groups (Table 3). However, the TECA score had no significant relationship with BDI-II score or WIS score. Permission must be obtained for use of copyrighted material from other sources (including the web). Please note that it is compulsory to follow figure instructions.

**Table 3:** Characteristics of patients with different TECA scores.

	TECA very low	TECA low	TECA medium	TECA High	TECA very high	*p*-value for trend
	(*n* = 10)	(*n* = 24)	(*n* = 36)	(*n* = 53)	(*n* = 31)	
Age (years)	41 ± 20	43 ± 17	35 ± 12	39 ± 13	43 ± 14	0.601
Gender (female)	4 (40.0%)	16 (66.7%)	32 (88.9%)	39 (73.6%)	25 (80.6%)	0.065
BDI-II	14.1 ± 5.0	13.4 ± 5.4	13.6 ± 5.5	12.4 ± 5.8	11.9 ± 6.0	0.142
Severe	0 (0.0%)	0 (0.0%)	1 (2.8%)	1 (1.9%)	0 (0.0%)	0.696
Mild	4 (40.0%)	5 (20.8%)	14 (38.9%)	16 (30.2%)	12 (38.7%)	
Moderate	2 (20.0%)	4 (16.7%)	3 (8.3%)	5 (9.4%)	2 (6.5%)	
No depression	4 (40.0%)	15 (62.5%)	18 (50.0%)	31 (58.5%)	17 (54.8%)	
WIS	2.9 ± 1.6	2.7 ± 1.5	2.9 ± 1.3	2.3 ± 1.5	2.7 ± 1.4	0.405
Permanent tiredness	7 (70.0%)	16 (66.7%)	19 (52.8%)	20 (37.7%)	17 (54.8%)	0.122
Infections and colds	4 (40.0%)	10 (41.7%)	12 (33.3%)	16 (30.2%)	17 (54.8%)	0.477
Slow healing	4 (40.0%)	4 (16.7%)	7 (19.4%)	11 (20.8%)	3 (9.7%)	0.145
Diarrhea	2 (20.0%)	6 (25.0%)	12 (33.3%)	12 (22.6%)	6 (19.4%)	0.588
Herpes	2 (20.0%)	11 (45.8%)	17 (47.2%)	18 (34.0%)	13 (41.9%)	0.878
Insomnia	5 (50.0%)	14 (58.3%)	21 (58.3%)	25 (47.2%)	18 (58.1%)	0.893
Dry eyes	5 (50.0%)	8 (33.3%)	16 (44.4%)	20 (37.7%)	11 (35.5%)	0.625
PEC, total	144 ± 18	154 ± 28	173 ± 30	191 ± 20	201 ± 25	<0.001
Low	9 (90.0%)	15 (62.5%)	16 (44.4%)	7 (13.2%)	5 (16.1%)	<0.001
Medium	1 (10.0%)	5(20.8%)	12 (33.3%)	22 (41.5%)	4 (12.9%)	
High	0 (0.0%)	1 (4.2%)	8 (22.2%)	20 (37.7%)	22 (71.0%)	
PEC, intrapersonal	71 ± 15	79 ± 16	87 ± 22	96 ± 17	101 ± 19	<0.001
Low	8 (80.0%)	15 (62.5%)	15 (41.7%)	11 (20.8%)	7 (22.6%)	<0.001
Medium	2 (20.0%)	4 (16.7%)	12 (33.3%)	18 (34.0%)	6 (19.4%)	
High	0 (0.0%)	2 (8.3%)	9 (25.0%)	20 (37.7%)	18 (58.1%)	
PEC, interpersonal	73 ± 11	75 ± 18	91 ± 20	95 ± 53	100 ± 31	<0.001
Low	9 (90.0%)	15 (62.5%)	15 (41.7%)	6 (11.3%)	2 (6.5%)	<0.001
Medium	1 (10.0%)	3 (12.5%)	11 (30.6%)	18 (34.0%)	9 (29.0%)	
High	0 (0.0%)	3 (12.5%)	9 (25.0%)	25 (47.2%)	20 (64.5%)	
TECA, total	2 ± 1	21 ± 6	45 ± 9	80 ± 7	97 ± 2	<0.001
TECA, Adoption perspectives (AP)	6 ± 12	27 ± 26	44 ± 24	67 ± 25	78 ± 26	<0.001
TECA, Emotional understanding (CE)	19 ± 26	38 ± 29	53 ± 24	79 ± 21	89 ± 14	<0.001
TECA, Empathic stress (EE)	11 ± 11	26 ± 25	42 ± 25	56 ± 28	78 ± 22	<0.001
TECA, Empathic joy (AE)	14 ± 14	31 ± 26	63 ± 30	77 ± 19	84 ± 17	<0.001

Values are expressed as mean ± SD or *n* (%). The *p*-value for trend was calculated using the TECA score as a continuous variable in the correlation analysis.

### 3.3. Correlations of BDI-II, WIS, TECA, and PEC scores

We then performed linear regression analysis to calculate the correlations of all the different psychometric scores (Table 4). These results demonstrated that the BDI-II score had a positive correlation with the WIS score, but negative correlations with the total PEC score and the intrapersonal PEC score. The WIS score had negative correlations with the total PEC score and intrapersonal PEC score. The total TECA score had positive correlations with the total PEC score, intrapersonal PEC score, and interpersonal PEC score. The total PEC score had positive correlations with the intrapersonal PEC score and the interpersonal PEC score. The intrapersonal PEC score had a positive correlation with the interpersonal PEC score.

**Table 4:** Linear correlations of scores for BDI-II, WIS, total TECA, total PEC, intrapersonal PEC, and interpersonal PEC.

		BDI-II	WIS	Total TECA	Total PEC	Intrapersonal PEC
BDI-II						
Crude						
Adjusted model^#^						
WIS						
Crude		0.43 (0.30–0.54)^***^				
Adjusted model^#^		0.42 (0.30–0.53)^***^				
Total TECA						
Crude		−0.10 (−0.25–0.05)	−0.09 (−0.25–0.08)			
Adjusted model^#^		−0.11 (−0.26–0.04)	−0.12 (−0.27–0.06)			
Total PEC						
Crude		−0.31 (−0.44–−0.17)^***^	−0.27 (−0.39–−0.10)^***^	0.60 (0.49–0.69)^***^		
Adjusted model^#^		−0.33 (−0.46–−0.19)^***^	−0.29 (−0.42–−0.14)^***^	0.60 (0.49–0.69)^***^		
Intrapersonal PEC						
Crude		−0.38 (−0.50–−0.25)^***^	−0.30 (−0.42–−0.17)^***^	0.44 (0.30–0.56)^***^	0.88 (0.84–0.92)^***^	
Adjusted model^#^		−0.40 (−0.52–−0.27)^***^	−0.33 (−0.45–−0.20)^***^	0.43 (0.28–0.56)^***^	0.88 (0.83–0.91)^***^	
Interpersonal PEC						
Crude		−0.07 (−0.24–0.07)	−0.05 (−0.25–0.13)	0.51 (0.33–0.69)^***^	0.59 (0.24–0.83)^***^	0.35 (0.19–0.51)^***^
Adjusted model^#^		−0.08 (−0.24–0.06)	−0.07 (−0.25–0.11)	0.51 (0.31–0.68)^***^	0.59 (0.22–0.83)^***^	0.35 (0.17–0.51)^***^

Results are expressed as β coefficients (95% CIs) for the crude and adjusted models.

# Adjusted for age and gender.

*** *p* < 0.001.

### 3.4. Discriminant analysis, ROC analysis, and logistic regression analysis

All variables with a *p* < 0.05 in univariate analysis (Table 1) were entered into a stepwise discriminant analysis to obtain a final model for discrimination between individuals with and without depression. The final model showed that Wilks’ lambda, as a test of discriminant function, was highly significant (λ = 0.837; χ^2^ = 25.6 df = 1, *p* < 0.001) and the only selected variable was WIS with a canonical discriminant coefficient of 0.763.

**Figure 1:**
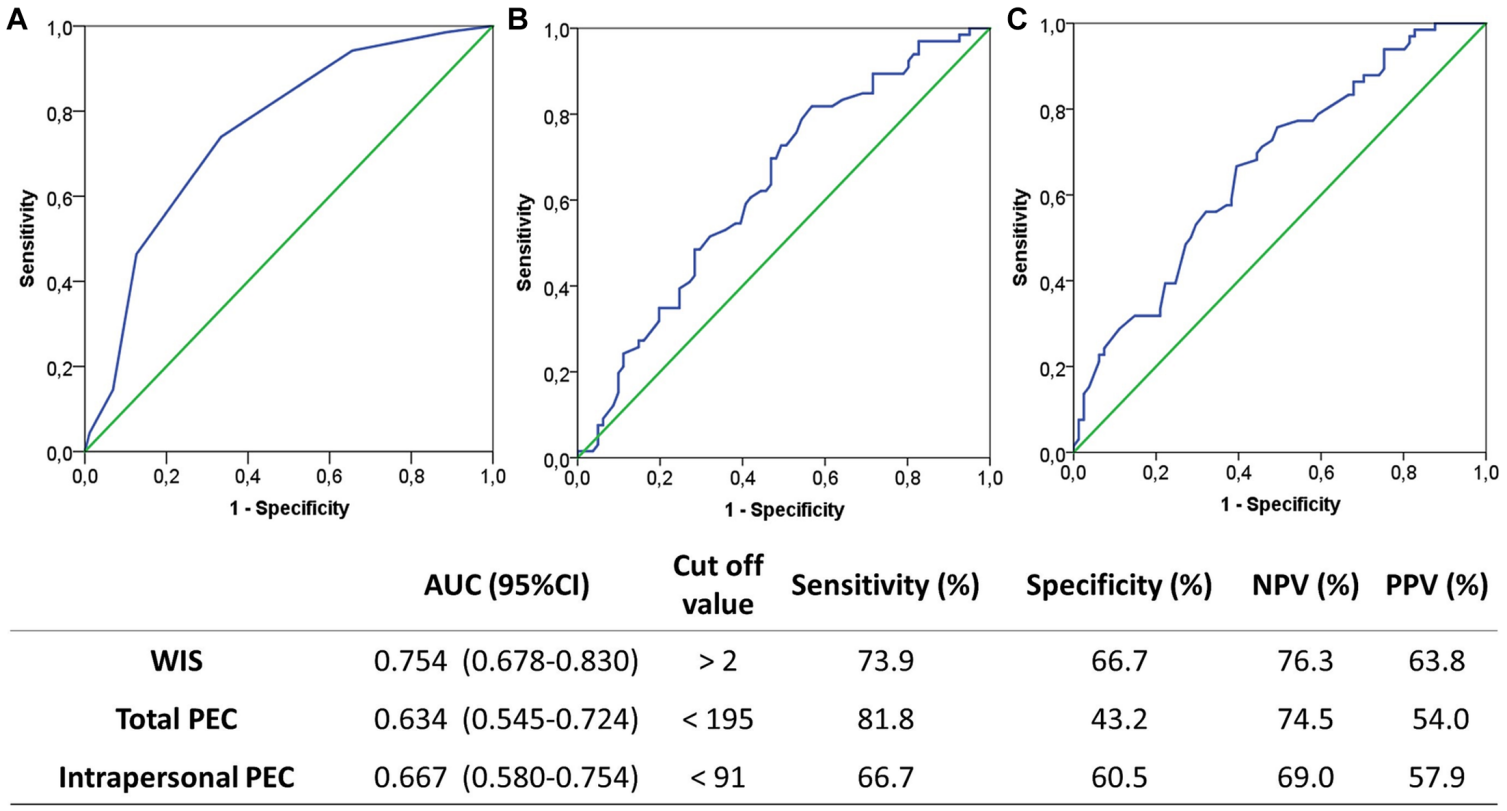
Receiver operating characteristic analysis for prediction of depression based on WIS score **(A)**, total PEC score **(B)**, and intrapersonal PEC score **(C)**. Optimal cutoff values were determined by the Youden index (J), defined as sensitivity + specificity −1.

We then used ROC analysis to examine use of WIS score, total PEC score, and intrapersonal PEC score for prediction of depression (Figures 1A–C). The WIS score had an area under the curve (AUC) of 0.754, optimal cutoff of 2, sensitivity of 73.9%, and specificity of 66.7%. Thus, the WIS score had a higher AUC than the total PEC score (0.634) and the intrapersonal PEC score (0.667), and better specificity than the total PEC score (43.2%) and intrapersonal PEC score (60.5%). However, the total PEC score had the highest sensitivity (81.8%).

**Figure 2:**
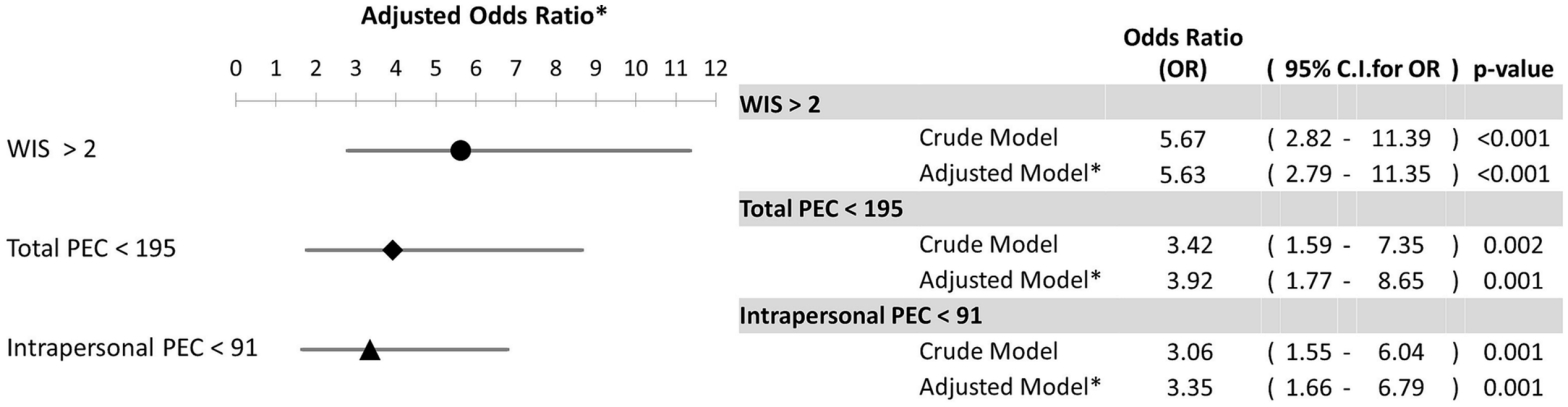
Relationship of depression with WIS score, total PEC score, and intrapersonal PEC score, based on optimal cutoff values from the ROC analysis. ^*^Model was adjusted by age and gender.

Finally, we performed univariate and multivariate logistic regression analysis to calculate the crude and adjusted ORs for the association of WIS score, total PEC score, and intrapersonal PEC score with depression, using cut-off values from the ROC analysis (Figure 2). In the crude and adjusted models, each of these three psychometric scores had significant associations with depression. After adjustment for age and sex, the WIS score had a stronger relationship with depression (aOR = 5.63; 95%CI =2.79–11.35) than the total PEC score (aOR = 3.92; 95%CI = 1.77–8.65) or the intrapersonal PEC score (aOR = 3.34, 95%CI = 1.66–6.79). According to these values, patients with WIS values greater than 2 were 5.63 times more likely to present depression in comparison to those with WIS values lower than this value. Also, patients with total PEC value lower than 195 are 3.92 times more likely to have depression compared to those with PEC values greater than 195. Furthermore, when multivariate logistic regression analysis using stepwise method were performed, the obtained final model showed that the factors associated to depression were both WIS>2 [OR = 4.24; 95%CI = 2.04–8.78] and total PEC < 195[OR = 2.69; 95%CI = 1.19–6.06]. The Hosmer-Lemeshow goodness of fit test (χ^2^ = 2.495; df = 2; *p* = 0.287) indicated a very good fit of this model.

## 4. Discussion

Previous studies have documented positive relationships of depression with high oxidative stress (Bouayed et al., 2009; Scapagnini et al., 2012) and consumption of certain food groups (Lai et al., 2014; Rahe et al., 2014; Grases et al., 2019), and a close relationship between nutrition and the immune system (Nova et al., 2002; Dantzer et al., 2008). There is also evidence of a relationship of emotional intelligence with depression (Schutte et al., 2007; Martins et al., 2010), and several studies showed that the ability of an individual to regulate his or her own emotional state was associated with fewer diseases (Goldman et al., 1996; Fernández-Berrocal et al., 2003). However, the relationship between empathy and depression is not so clear. For example, some researchers found correlations of depression with cognitive empathy and affective empathy (Tone and Tully, 2014), but other studies only reported a relationship between affective empathy and depression (Gambin and Sharp, 2018; Calandri et al., 2019) or between cognitive empathy and depression (Bennik et al., 2019).

The presence of a weakened immune system can be estimated by measuring the presence of numerous pathologies, such as infections and frequent colds, permanent fatigue, slow wound healing, persistent and recurrent diarrhea, recurrent herpes, insomnia and difficulty sleeping, and dry eyes. In the present study, we used these seven criteria to develop a WIS scale. Notably, nutritional imbalances and other factors related to nutrition weaken the immune system and contribute to these pathologies. Oxidative stress is also associated with the pathogenesis of many diseases, some of which are related to psychiatric disorders (Halliwell, 2006; Bouayed et al., 2009; Hovatta et al., 2010; Grases et al., 2014), including depression (Maes et al., 2011; Kovacic and Somanathan, 2012; Scapagnini et al., 2012). Our finding of a relationship between depression and a weakened immune system supports these previous findings. Our finding of a correlation of intrapersonal emotional competence with depression could be because an individual with a high level of emotional competence is less likely to develop mental disorders, including depression (Aldao and Nolen-Hoeksema, 2010; Nolidin et al., 2013; Persich et al., 2021).

However, we also found that empathy had no correlation with depression or with a weakened immune system. Empathy is a component of an individual’s overall emotional intelligence and refers to the ability to understand and comprehend the emotions of others. An appropriate level of empathy enables a person to have healthy relationships with others. Thus, on the one hand empathy could be a benefit because it provides an individual with a better social support network, which is particularly important when the individual has a depressed mood. On the other hand, an excess of empathy could be detrimental if an individual develops intense worrying about the problems of others. The role of empathy in depression is therefore complex. Therefore, preventing or reducing depression does not simply depend on the ability of an individual to understand other people and thereby develop improved social relationships; instead, an important step in preventing or reducing depression depends on an individual’s ability to regulate his or her own emotions. Having the skills to recognize and manage one’s own emotions can protect against depressed moods, and this has obvious repercussions for the immune system. This interpretation emphasizes the importance of preventive measures that improve emotional competences at the intrapersonal level as a method to improve mental and physiological health. Our study also demonstrated that empathy was associated with emotional competences. Obviously, empathy is a component of emotional intelligence, and several studies showed a correlation between emotional intelligence and empathy (Bertram et al., 2016; Abe et al., 2018).

The relationship of depression with poor nutrition (Lai et al., 2014; Rahe et al., 2014; Grases et al., 2019) could partly explain its correlation with immune-based diseases, because there are close links of poor nutritional status, a weakened immune system, and mild depression. When major depression occurs, the underlying mechanisms are probably more complex, and there is evidence that the excessive production of pro-inflammatory cytokines can affect brain function and lead to psychiatric disorders (Dantzer et al., 2008; Miller and Raison, 2016; Ramírez et al., 2018; Branchi et al., 2021). These previous findings are in agreement with the results of the present study, which found a relationship between intrapersonal emotional competence and a weakened immune system, and a correlation between emotional competences and depression. However, just as we identified no correlation between empathy and a weakened immune system, we also found no correlation between empathy and depression. In fact, as is commented in the Results section, multivariate logistic regression analysis showed that the factors associated to depression were both, WIS and total PEC.

Our study has some limitations. First, the sample size was small, and it was a cross-sectional study performed at a single medical center. Therefore, our findings should be interpreted with caution and may not be generalizable to other populations. Also, even though we found that the WIS score, total PEC score, and interpersonal PEC score were associated with depression, these associations do not necessarily imply causality. Prospective multicenter longitudinal studies are needed to fully clarify how these factors contribute to the genesis and progression of depression. It would be interesting to extend these studies with a larger number of individuals that would allow for the evaluation of different age groups, since the results obtained could vary according to each group. The relationship that exists between emotional intelligence and depression reinforces the idea that education in emotional intelligence development can be very important to avoid depression. It would be also important to carry out a longitudinal study to assess whether improving emotional competences could be a protective element in preventing depression. The study would serve to guide the educational skills that should be worked on at school, with the aim of providing students with life skills.

## 5. Conclusion

We found that emotional intelligence (intrapersonal emotional competences) correlated with depression, and that emotional intelligence and depression were associated with symptoms of a weakened immune system. However, empathy had no correlation with depression or with symptoms of a weakened immune system. These findings reinforce the view that interventions for individuals with low moods should seek to improve their intrapersonal emotional skills, and that these interventions could also provide benefits to the immune system. The relationship between a depressed mood and interpersonal skills (empathy) is more complex and needs further study.

## Data availability statement

The raw data supporting the conclusions of this article will be made available by the authors, without undue reservation.

## Ethics statement

The studies involving humans were approved by local Ethics Committee of the hospital (Psychology Center, Juaneda Group Hospitals). The studies were conducted in accordance with the local legislation and institutional requirements. The participants provided their written informed consent to participate in this study.

## Author contributions

GG, MC, and FG contributed to conception and design of the study. GG organized the database. PS performed the statistical analysis. GG, MC, FG, and PS wrote the first draft of the manuscript and wrote sections of the manuscript. All authors contributed to the article and approved the submitted version.
