# Supplementary material for: Relationship of depression with empathy, emotional intelligence, and symptoms of a weakened immune system
Source: Front Psychol. 2023 Oct 27;14:1250636. doi: 10.3389/fpsyg.2023.1250636 (PMC10641773; doi:10.3389/fpsyg.2023.1250636)
Supplement: Supplementary file 1 [file Data_Sheet_1.docx]

Supplementary Material

Relationship of depression with empathy, emotional intelligence, and symptoms of a weakened immune system

Grases G.^1^, Colom M.A.^2^, Sanchis P.^3,*^, Grases, F.^3^

*** Correspondence:** Pilar Sanchis: pilar.sanchis@uib.es

# Supplementary Tables

**Supplementary Table 1.**

*TECA scale.*

| **Percentile score** | **Direct score** | **Interpretation** |
| --- | --- | --- |
| 94 to 99 | 66 or higher | Extremely high score, which indicates decision making is more difficult and the presence of a more emotionally charged state |
| 70 to 93 | 56 to 65 | High empathy |
| 31 to 69 | 45 to 55 | Medium empathy |
| 7 to 30 | 35 a 44 | Low empathy, which implies little flexibility and denotes little understanding of others, making communication with people difficult |
| 1 to 29 | 34 or lower | Extremely low empathy, which implies a significant communication deficit |

**Supplementary Table 2.**

*PEC scale.*

|  | Low emotional competence | Medium emotional competence | High emotional competence |
| --- | --- | --- | --- |
| Intrapersonal | ≤ 84 | 85-99 | ≥ 100 |
| Interpersonal | ≤ 83 | 84-95 | ≥ 96 |
| Total | ≤ 169 | 170-192 | ≥ 193 |
